# Examination of IgG Fc Receptor CD16A and CD64 Expression by Canine Leukocytes and Their ADCC Activity in Engineered NK Cells

**DOI:** 10.3389/fimmu.2022.841859

**Published:** 2022-02-24

**Authors:** Robert Hullsiek, Yunfang Li, Kristin M. Snyder, Sam Wang, Da Di, Antonella Borgatti, Chae Lee, Peter F. Moore, Cong Zhu, Chiara Fattori, Jaime F. Modiano, Jianming Wu, Bruce Walcheck

**Affiliations:** ^1^ Department of Veterinary and Biomedical Sciences, University of Minnesota, St. Paul, MN, United States; ^2^ Animal Cancer Care and Research Program, University of Minnesota, St. Paul, MN, United States; ^3^ Department of Veterinary Clinical Sciences, College of Veterinary Medicine, University of Minnesota, St. Paul, MN, United States; ^4^ Masonic Cancer Center, University of Minnesota, Minneapolis, MN, United States; ^5^ Center for Immunology, University of Minnesota, Minneapolis, MN, United States; ^6^ Clinical Investigation Center, University of Minnesota, St. Paul, MN, United States; ^7^ Department of Pathology, Microbiology, Immunology, School of Veterinary Medicine, University of California, Davis, CA, United States; ^8^ Stem Cell Institute, University of Minnesota, Minneapolis, MN, United States; ^9^ Institute for Engineering in Medicine, University of Minnesota, Minneapolis, MN, United States; ^10^ Department of Laboratory Medicine and Pathology, School of Medicine, University of Minnesota, Minneapolis, MN, United States

**Keywords:** natural killer cells (NK cells), Fc receptor, IgG, canine (dog), antibody-dependent cell-mediated cytotoxicity (ADCC)

## Abstract

Human natural killer (NK) cells can target tumor cells in an antigen-specific manner by the recognition of cell bound antibodies. This process induces antibody-dependent cell-mediated cytotoxicity (ADCC) and is exclusively mediated by the low affinity IgG Fc receptor CD16A (FcγRIIIA). Exploiting ADCC by NK cells is a major area of emphasis for advancing cancer immunotherapies. CD64 (FcγRI) is the only high affinity IgG FcR and it binds to the same IgG isotypes as CD16A, but it is not expressed by human NK cells. We have generated engineered human NK cells expressing recombinant CD64 with the goal of increasing their ADCC potency. Preclinical testing of this approach is essential for establishing efficacy and safety of the engineered NK cells. The dog provides particular advantages as a model, which includes spontaneous development of cancer in the setting of an intact and outbred immune system. To advance this immunotherapy model, we cloned canine CD16A and CD64 and generated specific mAbs. We report here for the first time the expression patterns of these FcγRs on dog peripheral blood leukocytes. CD64 was expressed by neutrophils and monocytes, but not lymphocytes, while canine CD16A was expressed at high levels by a subset of monocytes and lymphocytes. These expression patterns are similar to that of human leukocytes. Based on phenotypic characteristics, the CD16A^+^ lymphocytes consisted of T cells (CD3^+^ CD8^+^ CD5^dim^ α/β TCR^+^) and NK cells (CD3^−^ CD5^−^ CD94^+^), but not B cells. Interestingly, the majority of canine CD16A^+^ lymphocytes were from the T cell population. Like human CD16A, canine CD16A was downregulated by a disintegrin and metalloproteinase 17 (ADAM17) upon leukocyte activation, revealing a conserved means of regulation. We also directly demonstrate that both canine CD16A and CD64 can induce ADCC when expressed in the NK cell line NK-92. These findings pave the way to engineering canine NK cells or T cells with high affinity recombinant canine CD64 to maximize ADCC and to test their safety and efficacy to benefit both humans and dogs.

## Introduction

NK cells are innate cytotoxic lymphocytes that interrogate cells in the body to identify those that are stressed, infected, or neoplastic (1). NK cells are rapidly activated and release cytolytic factors as well as cytokines and chemokines that stimulate other components of the immune system. NK cell activation is mediated by various ligands and by antibodies attached to target cells (1). The latter process induces an effector function referred to as antibody-dependent cell-mediated cytotoxicity (ADCC) and it is exclusively mediated by the IgG Fc receptor CD16A (FcγRIIIA) on human NK cells (2, 3).

Anti-tumor mAbs provide a rapidly expanding repertoire of antigen-specific targeting elements for NK cells (4). However, their clinical performance is limited by certain attributes of CD16A. It is well described that CD16A undergoes rapid ectodomain shedding by a disintegrin and metalloproteinase 17 (ADAM17) upon NK cell activation with diverse stimuli (3). Preventing this process in human NK cells by engineering a noncleavable CD16A or blocking ADAM17 enhanced their release of IFNγ and target cell killing in the presence of mAb therapies (5–7). CD16A is also a low affinity FcγR that stably binds to cell-bound IgG, but not soluble monomeric IgG. In humans, two CD16A allelic variants with either a phenylalanine (F) or a valine (V) at amino acid position 158 have been described (8). CD16A-158V has ≈ 2-fold higher affinity for IgG1 as compared to CD16A-158F (9, 10). Studies have shown that cancer patients homozygous for CD16A-158V responded significantly better to tumor targeting mAbs (11–13), indicating that increased binding affinity between CD16A and tumor-targeting mAbs will enhance NK cell anti-tumor effector functions. A strategy to increase both the binding affinity and avidity between NK cells and antibody-opsonized tumor cells has involved modifying the FcγR on NK cells (3, 14, 15). Human CD64 (FcγRI), the only high affinity IgG Fc receptor, binds to the same IgG isotypes as CD16A but with > 30-fold higher affinity than CD16A-158V (10, 16). CD64, however, is expressed by myeloid leukocyte populations but not lymphocytes, including NK cells (10). Therefore, we have engineered human NK cells to express recombinant versions of CD64 to increase their attachment efficiency to antibody-coated tumor cells to kill these cells (17, 18). Moreover, due to its high affinity state, NK cells expressing recombinant CD64 can be “armed” with anti-tumor mAbs, which can be switched for universal tumor antigen targeting (14, 17, 18).

The clinical translation of engineered NK cell immunotherapies into successful cancer therapies has been slow, due in part to the use of animal models with critical species differences in their immune cell effector functions. For instance, the study of ADCC in mice is confounded by the considerable divergence in human and mouse FcγR expression profiles and function. Mature human NK cells uniformly express high levels of CD16A under steady state conditions. Mouse leukocytes express two versions of CD16 (10). Mouse CD16 (FcγRIII) is expressed at low levels by NK cells and is actually more closely related to human CD32A (FcγRIIA), which is not expressed by human NK cells (10, 19). CD16-2 (FcγRIV) is the mouse orthologue of CD16A, but it is not expressed by resting NK cells (20). In addition, CD16-2 has been reported to bind IgE and promote IgE-mediated inflammation (21, 22). Both versions of mouse CD16 are also not shed by ADAM17 (23), demonstrating differences in their regulation compared to human CD16A. An ideal animal model for understanding the mechanisms that underlie success and failure of human immunotherapies should incorporate heterogeneous spontaneous disease and an intact immune system that is similar to humans. A comparable incidence of human and certain canine malignancies, combined with their shared biologic and pathologic characteristics, and similar response to therapy indicate that dogs can provide a clinically relevant disease model (24–26).

Based on morphology, canine NK cells are medium to large lymphocytes with electron-dense intracytoplasmic granules that contain granzyme B and perforin. These cells express at the mRNA level several genes associated with NK cells, such as NK1.1, NKG2D, CD94, CD96, NKp30, NKp44, NKp46, NKG2D, CD16A, DNAM-1, perforin, and granzyme B (26, 27), and based on transcriptome analysis, canine NK cells are globally more similar to human NK cells than to mouse NK cells (26). Canine NK cells also mediate natural cytotoxicity and ADCC (27–32). However, a lack of available species-specific antibodies has hindered efforts to study FcγRs on canine leukocytes. The focus of our study was to characterize canine CD16A and CD64 leukocyte expression patterns and their capacity to induce ADCC.

## Materials and Methods

### Cells

Peripheral blood was collected from healthy pet dogs with consent from owners. The dogs consisted of various breeds and mixed breeds. All animals had received routine veterinary care, vaccinations, parasite control, and were considered to be in overall good health. Blood collection was carried out in strict accordance with the recommendations in the Guide for the Care and Use of Laboratory Animals of the National Institutes of Health. The protocol was approved by the Institutional Animal Care and Use Committee of the University of Minnesota (Protocol Numbers: 1304-30546A and 1903–36913A). Blood was collected in K2-EDTA blood collection tubes (BD Biosciences, Franklin Lakes, NJ). Total leukocytes and PBMCs were isolated as we have previously described (33). Leukocytes with ≥ 95% viability, as assessed by trypan blue staining, were used in the described assays. Cell lines used were NK-92MI cells, an IL-2 independent version of NK-92 cells (34), SKOV-3 cells, and 293T, which were obtained from ATCC (Manassas, VA). FreeStyle 293-F cells were obtained from Thermo Fisher Scientific (Waltham, MA). All cells were cultured per the manufacturer’s directions, as we have previously described (17, 18). For cell activation, leukocytes were stimulated with phorbol-12-myristate-13-acetate (PMA) (MilliporeSigma, Burlington, MA), as previously described (33). Some cells were pre-incubated for 30 min on ice with the function-blocking ant-ADAM17 mAb MEDI3622 (5 μg/ml) prior to activation.

### Antibodies

To produce mAbs to canine CD16A and CD64, BALB/c mice were immunized intraperitoneally with purified soluble protein of each FcγR and hybridomas generated based on previous methods (35, 36). The anti-ADAM17 mAb MEDI3622 has been previously described (5, 33, 37). The anti-canine α/β TCR and γ/δ TCR mAbs (CA15.8G7 and CA20.8H1, respectively) have been previously described (30, 38, 39). Isotype-matched negative control mAbs were purchased from BioLegend (San Diego, CA) and BD Biosciences (Franklin Lakes, NJ). Affinity purified canine serum IgG was purchased from Southern Biotech (Birmingham, AL). Fluorophore or biotin-conjugated F(ab′)_2_ goat anti-mouse secondary antibodies and fluorophore-conjugated streptavidin were purchased from Jackson ImmunoResearch Laboratories (West Grove, PA) and BioLegend. Zombie Violet™ Fixable Viability Kit was purchased from Biolegend. All commercially available mAbs are listed in **Table 1**.

**Table 1:** Commercially available mAbs.

Antigen	Clone	Species	Company
Influenza Hemagglutinin A-Tag	12CA5	Mouse	Santa Cruz Biotechnology, Dallas, TX
canine CD4	YKIX302.9	Rat	Bio-Rad Laboratories, Hercules, CA
canine CD3	CA17.2A12	Mouse	Bio-Rad Laboratories
canine CD5	YKIX322.3	Rat	Bio-Rad Laboratories
human CD14	TÜK4	Mouse	Bio-Rad Laboratories
canine CD20	6C12	Caninized	Invivogen, San Diego, CA
canine CD20	6C12	Mouse	Invivogen
canine NKp46	48A	Mouse	MilliporeSigma, Burlington, MA
human CD94	HP-3D9	Mouse	BD Biosciences, Franklin Lakes, NJ

### Flow Cytometry

For cell staining, nonspecific antibody binding sites were blocked for 30 min using 25% canine serum and 25% FBS in PBS buffer (without Ca^+2^ and Mg^+2^) (Lonza, Walkersville, MD) prior to their staining with antibodies. All cell staining was analyzed on a FACSCelesta instruments (BD Biosciences), as previously described (33). Briefly, for controls, fluorescence minus one was used as well as appropriate isotype-matched antibodies. An FSC-A/SSC-A plot was used to set an electronic gate on leukocyte populations and an FSC-A/FSC-H plot was used to set an electronic gate on single cells. Fixable viability dyes eFluor 506 (Thermo Fisher Scientific) or Zombie Violet (BioLegend, San Diego, CA) were used to assess live vs. dead cells, as per the manufacturer’s instructions. Canine leukocyte subsets were identified based on their forward and side light-scattering characteristics and various phenotypic markers, as described. In some cases, we attempted to enhance the staining by particular mAbs to better distinguish leukocyte subsets, as was the case for the anti-CD94 mAb HP-3D9. This was done by staining dog cells leukocytes with an unconjugated mAb, biotin-conjugated F(ab′)_2_ goat anti-mouse secondary antibodies, then fluorophore-conjugated streptavidin. Cell washes were performed between all steps. If the cells were to be stained with additional mouse-derived mAbs, the cells were first treated for 15 min with 5% mouse serum in PBS buffer. This served to occupy any free arms of cell-attached anti-mouse secondary antibodies, preventing them from binding subsequently added mouse-derived mAbs, which would result in artifactual staining.

Canine IgG adsorption to NK-92 cells was performed as previously described (17, 18), with some modifications. Briefly, cells were incubated with the indicated concentrations of canine IgG previously biotinylated using an EZ-Link™ Sulfo-NHS-LC-Biotin Kit (Thermo Fisher Scientific) per the manufacturer’s instructions, for 2 h at 37°C in MEM-α basal media supplemented with HEPES (10 mM), and 2-mercaptoethanol (0.1 mM). Binding levels of biotinylated canine IgG was determined by staining the cells with allophycocyanin-streptavidin (Jackson ImmunoResearch).

### Western Blotting

Protein concentrations were quantified using a bicinchoninic acid assay (BCA) (Pierce Biotechnology, Waltham, MA). Five micrograms of protein [1X Laemmli sample buffer (Bio-Rad Laboratories), 0.1M DTT] was resolved by SDS-PAGE and transferred to a nitrocellulose membrane. Blots were blocked in Intercept TBS blocking buffer (LI-COR Biosciences, Lincoln, NE) for 1 h at room temperature and incubated with primary antibodies and Quick Western IRDye 680RD detection reagent (LI-COR Biosciences) overnight at 4°C. Blots were visualized using an Odyssey imager (LI-COR Biosciences). Primary antibodies used were anti-canine CD64 clone 10 and anti-canine-CD16A clone 4A5 at 5μg/ml each.

### Cytotoxicity Assays

ADCC assays were conducted using a DELFIA EuTDA cytotoxicity according to the manufacturer’s instructions (PerkinElmer, Waltham, MA) and as we have previously described (17, 18). Briefly, SKOV-3-canine CD20 target cells were labeled with Bis(acetoxymethyl)-2-2:6,2 terpyridine 6,6 dicarboxylate (BATDA) for 30 min in their culture medium, washed in culture medium, and pipetted into a 96-well non-tissue culture-treated U-bottom plates at a density of 8 × 10^3^ cells/well. Caninized anti-canine CD20 mAb was either adsorbed to NK-92 cells at 5 μg/ml in MEM-α basal media supplemented with HEPES (10 mM) then washed with MEM-α basal media or the mAb was added directly to the SKOV-3 cells at 5 μg/ml and NK-92 cells were added at the indicated E:T ratios. The plates were centrifuged at 400 × g for 1 min and then incubated for 2 h in a humidified 5% CO_2_ atmosphere at 37°C. At the end of the incubation, the plates were centrifuged at 500 × g for 5 min and supernatants were transferred to a 96 well DELFIA Yellow Plate (PerkinElmer) and combined with europium. Fluorescence was measured by time-resolved fluorometry using a BMG Labtech CLARIOstar plate reader (Cary, NC). BATDA-labeled target cells alone with or without therapeutic antibodies were cultured in parallel to assess spontaneous lysis and in the presence of 1% Triton-X to measure maximum lysis. ADCC for each sample is represented as % specific release and was calculated using the following formula: Percent Specific Release = (Experimental release – Spontaneous release)/(Maximal release – Spontaneous release)*100. For each experiment, assays were conducted in triplicate that were measured using two or three replicate assay wells.

### Cloning of Canine CD16A, CD64, and CD20, Generation of Expression Constructs, and Cell Line Transduction

Total RNA was isolated from canine peripheral blood leukocytes using TRIzol total RNA isolation reagent (Thermo Fisher Scientific). Peripheral blood cDNA was synthesized with the SuperScript First-Strand Synthesis (Thermo Fisher Scientific) and used in RT-PCR for expression construct generation. Full-length canine CD16A cDNA corresponding to two extracellular domains, transmembrane segment, and cytoplasmic region was amplified using the forward primer 5’-CTC TAG ACT GCC GGA TCC GCA GTG ACT TGC TGA CCC TAA TGT G-3’ and the reverse primer 5’-TCG AAT TTA AAT GGA TCC AGA GAG GTC CAG AGG GGT TGC TTT -3’. The underlined nucleotides indicate *Bam* HI restriction sites. To generate N-terminus hemagglutinin A (HA)-tagged canine CD16A, we amplified a cDNA fragment using the forward primer (5’-GCC CAG CCG GCC AGA TCT ACA CAA GCT GCA GAT GTC CCA-3’) and the reverse primer (5’- GCG GAT CCC GGG AGA TCT AGA GAG GTC CAG AGG GGT TGC TTT -3’). The underlined nucleotides indicate *Bgl* II restriction sites. An In-Fusion HD Cloning Kit (Takara Bio USA, San Jose, CA) was used to clone the canine CD16A cDNA fragment into a pDisplay vector (Thermo Fisher Scientific) linearized with *Bgl* II (New England Biolabs, Ipswich, MA). The expression cassette consisting of Igκ signal peptide, N-terminal HA tag, and canine CD16A was amplified using the forward primer (5’- TCT AGA CTG CCG GAT CCA CTA GTA ACG GCC GCC AGT GT-3’) and the reverse primer (5’- TCG AAT TTA AAT GGA TCC AGA GAG GTC CAG AGG GGT TGC TTT-3’). The underlined nucleotides indicate *Bam* HI restriction sites. Canine CD16A or HA-tagged CD16A were then cloned into the retrovirus expression vector pBMN-I-GFP (Addgene, Watertown, MA) linearized by *Bam* HI (New England Biolabs) using the In-Fusion HD Cloning Kit.

Full-length canine CD64 cDNA corresponding to three extracellular domains, transmembrane segment, and cytoplasmic region was amplified using the forward primer (5’-TCT AGA CTG CCG GAT CCG GAG ATA ACA TGT GGC TCT TGA CAG TTC TA -3’) and the reverse primer (5’- TCG AAT TTA AAT GGA TCC AAA AAG AAG TGG GAG GCA CCA TC-3’). The underlined nucleotides indicate *Bam* HI restriction sites. HA-tagged canine CD64 was amplified using the forward primer (5’-GCC CAG CCG GCC AGA TCT CAA ACA GAC CCC GTA AAG GCA -3’) and the reverse primer (5’- GCG GAT CCC GGG AGA TCT AAA AAG AAG TGG GAG GCA CCA TC -3’). The underlined nucleotides indicate *Bgl* II restriction sites. Their cloning into pDisplay and/or pBMN-I-GFP were carried out as described above.

Full-length canine CD20 cDNA was amplified using the forward primer (5’-TCT AGA CTG CCG GAT CCA GAG GGT GAG ATG ACA ACA CCC AGA-3’) and the reverse primer (5’-TCG AAT TTA AAT GGA TCC TTA AGG GAT GCT GTC GTT TTC TAT-3’). The underlined nucleotides indicate *Bam* HI restriction sites. The cloning of canine CD20 into pBMN-I-GFP was carried out as described above. The expression of all constructs in pBMN-I-GFP were confirmed using the sequencing primers 5’-TAG CTG GAA GAA CAC GCC CGT A-3’ and 5’-GCA GAA GTA GGA GCC ATT GTG T-3’. Pseudo retrovirus particles were generated as previously described (40), and were subsequently used for NK-92 or SKOV-3 cell transduction. Cells were sorted through FACSAria II cell sorting on GFP expression (BD Biosciences).

### Cloning of Soluble Canine CD16A and CD64, Generation of Expression Constructs, and Cell Line Transduction

Canine CD16A cDNA corresponding to amino acids 1-205 with a 6×histidine-tag at the carboxyl terminus was amplified using the forward primer 5’-GAA GAC ACC GAC TCT AGA GCA GTG ACT TGC TGA CCC TAA TGT GA -3’ (the underlined nucleotides indicate an *Xba* I restriction site) and the reverse primer 5’-GTA GTC AGC CCG GGA TCC TTA ATG ATG ATG ATG ATG ATG GGG CCA GTG TGA AAG GAG TA-3’ (the underlined nucleotides indicate a *Bam* HI restriction site). Canine CD64 cDNA corresponding to amino acids 1-280 with a 6×histidine-tag at the carboxyl terminus was amplified using the forward primer 5’-GAC TCT AGA GGA GAT AAC ATG TGG CTC TTG ACA GTT CTA -3’ (the underlined nucleotides indicate an Xba I restriction sites and the reverse primer 5’-CCG GGA TCC TTA ATG ATG ATG ATG ATG ATG CAC TTG AAG CTC CAA CTC AGG G-3’ (the underlined nucleotides indicate a Bam HI restriction sites). Amplified cDNA was digested by *Xba* I and *Bam* HI then and cloned into a pLenti-3F vector (a gift from Dr. Fang Li’s lab, University of Minnesota, St Paul, MN) digested with the same restriction enzymes. The expression of all constructs in pLenti-3F were confirmed using the sequencing primers 5’-CAT GGG AAA GCA TCG CTA CGA A-3’ and 5’-TCA GAT TGA CCA CAT GCC CCT C-3’. Pseudo-lentiviral particles containing soluble canine CD16A or CD64 were generated using 293T cells and packaging vectors pMD2.G and pCMV-dR8.74psPAX2 (Addgene). Pseudo-lentiviral particles were transduced into the FreeStyle 293-F cells. The 293-F cell line was established under puromycin selection. The 293-F cells stably expressing soluble canine CD16A or CD64 were cultured in the FreeStyle™ 293 Expression Medium (Thermo Fisher Scientific) and cell culture supernatants were harvested when cell density reached 2.5×10^6^/ml. Soluble canine CD16A or CD64 were purified from cell culture supernatants using a two-step purification procedure. First step, Ni‐affinity chromatography. His-tagged proteins in cell culture supernatants were purified on a HisTrap HP His tag protein purification column (Cytiva, Marlborough, MA) according to the manufacture’s protocol. Second step, Fast protein liquid chromatography. Soluble canine CD16A and CD64 from the first step purification were injected into a Superdex 200 Increase 10/300 GL column (Millipore-Sigma) on an AKTA pure protein purification system (Cytiva). The purity of proteins was >95% as determined by SDS-PAGE.

### Statistical Analyses

Comparison between two groups was done using Student t test. Comparison between three or more groups was done using one-way ANOVA followed by Tukey honest significance *post hoc* test. Results are depicted as mean ± SD. The symbols used to represent the p values were as follows; **, P ≤ 0.01; ***, P ≤ 0.001; ****, P ≤ 0.0001.

## Results

### Generation of Anti-cCD16A and cCD64 mAbs

**Figure 1:**
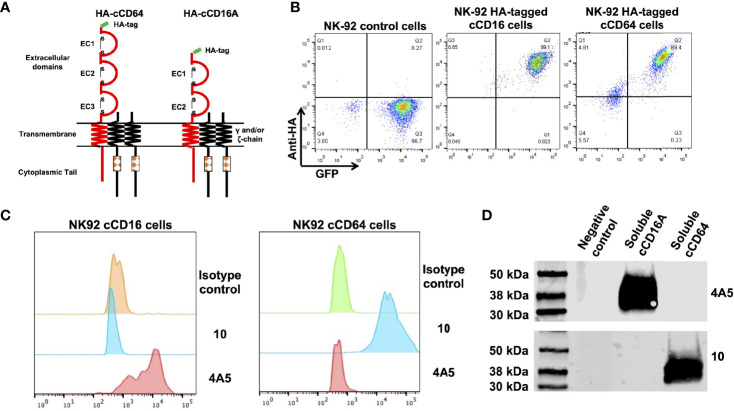
Characterization of anti-canine CD16A and CD64 mAbs. **(A)** Schematic representation of recombinant intact canine CD16A and CD64 with an N-terminus HA-tag. The signaling adaptors FcRγ and/or CD3ζ (γ and/or ζ chain) non-covalently associate with human CD16A and CD64 as a homo or heterodimer. **(B)** Flow cytometric analyses of NK-92 cells transduced with an empty vector (control cells), NK-92 canine CD16A (cCD16A) cells, and NK-92 canine CD64 (cCD64) cells stained with an anti-HA mAb. **(C)** Flow cytometric analyses of NK-92 cCD16A cells and NK-92 cCD64 cells stained with an isotype-matched negative control mAb, the anti-canine CD16A mAb 4A5, or the anti-canine CD64 mAb 10. **(D)** Western blot analysis of recombinant soluble canine CD16A, recombinant soluble canine CD64, or soluble human CD177 (negative control) using the mAbs 4A5 or 10. Equal protein loading was confirmed by BCA. All data are representative of three independent experiments.

In humans, CD16 consists of two isoforms, CD16A and CD16B, encoded by two highly homologous genes (41). CD16A is a transmembrane protein expressed by lymphocytes and some monocytes, whereas CD16B is linked to the plasma membrane *via* a GPI anchor and primarily expressed by neutrophils (42, 43). Canine CD16 is a transmembrane protein and therefore we refer to it here as CD16A (16). The CD16B isoform does not exist in the canine genome or cDNA (16, 19). Currently, there are no commercially available mAbs specific to canine CD16A or CD64 or any that are cross-reactive that we are aware of. For canine CD16A, this may be due to its relatively low levels of amino acid sequence identity and similarity with human CD16A (57.1% and 71.7%, respectively) (**Supplementary Figure 1A**). The amino acid sequence identity and similarity between canine CD64 and human CD64 are higher, 71.3% and 80.7%, respectively (**Supplementary Figure 1B**). Reactivity by the anti-human CD16 mAb clone LNK16 with dog peripheral blood monocytes has been reported (44), though others demonstrated a lack of specific activity by the same mAb with dog PBMCs (45). We also observed no specific reactivity by LNK16 and several other anti-human CD16 or CD64 mAbs with dog leukocytes (data not shown). Therefore, we expressed soluble forms of canine CD16A and CD64 to immunize mice and for initial hybridoma screening by ELISA. The anti-CD16A mAb clone 4A5 and the anti-CD64 mAb clone 10 were used in all analyses described below. Due to the lack of commercially available canine NK cell lines, we used the human NK cell line NK-92 for stable expression of intact versions of canine CD16A or CD64. An advantage of these cells is that they lack expression of endogenous FcγRs (46). The canine CD16A and CD64 expression constructs were engineered with an N-terminus HA-tag for detection, as illustrated in **Figure 1A**. The retroviral vector used for transduction also expressed eGFP as a separate protein for an additional marker (**Figure 1B**). As shown in **Figure 1C**, the anti-CD16A mAb clone 4A5 (IgG1) demonstrated selective reactivity with NK-92 canine CD16A cells and the anti-CD64 mAb clone 10 (IgG1) demonstrated selective reactivity with NK-92 canine CD64 cells. Neither mAb stained NK-92 control cells (**Figure 1C**). We also observed similar reactivity by 4A5 and 10 with recombinant soluble canine CD16A and CD64, respectively, by Western blotting (**Figure 1D**).

### Expression of CD16A and CD64 by Canine Leukocytes

**Figure 2:**
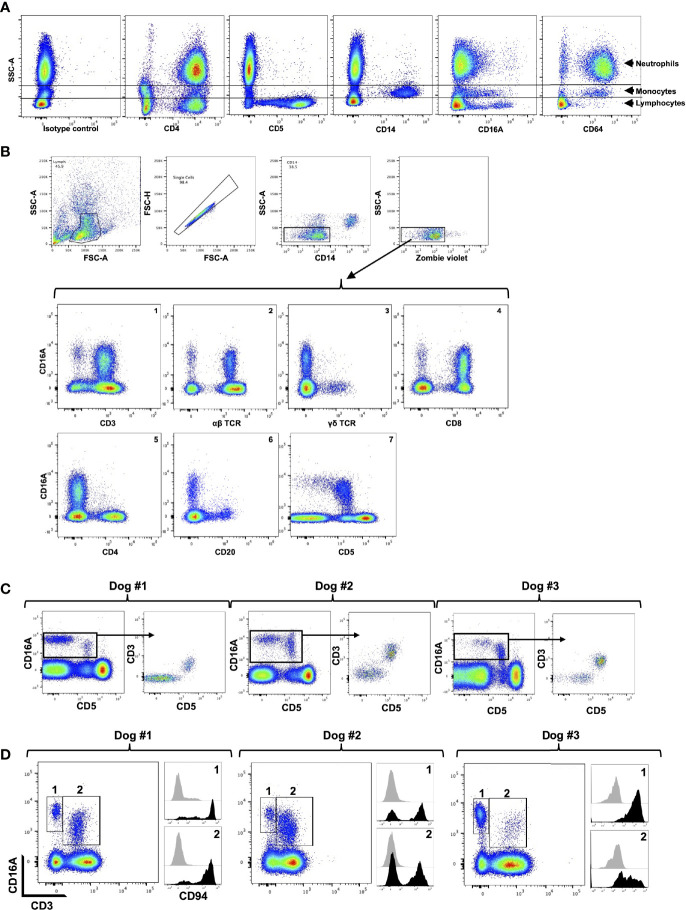
CD16A and CD64 expression by canine leukocyte subsets. **(A)** Canine peripheral blood leukocytes were stained for the indicated markers and examined by flow cytometry. The y-axis = light side scatter area (SSC-A) and the x-axis = Log 10 fluorescence. Data are representative of multiple independent experiments using leukocytes from separate canine donors. **(B)** CD16A is expressed by T cells and non-B cell, non-T cell lymphocytes. The top panels show the gating strategy on peripheral blood mononuclear cells to examine single cell, viable, lymphocytes. The gating strategy was used in B-C. The bottom panels show the expression of CD16A on various lymphocyte populations using the indicated markers. All data are representative of multiple independent experiments using leukocytes from separate canine donors. **(C)** CD16A expression on CD5^−^ and CD5^+^ lymphocytes. For each dog examined, CD16A versus CD5 staining of lymphocytes was determined (left panel). From this plot, CD16^+^ cells were gated and their expression of CD3 versus CD5 determined. **(D)** Expression of CD94 by CD3^−^ CD16^+^ and CD3^+^ CD16^+^ lymphocytes. These populations were gated on for each dog examined (left panel) and their staining levels of CD94 were determined (right panel), as indicated by the corresponding numbers. The black filled histograms represent CD94 staining, and the grey filled histograms represent isotype-matched negative control staining. For panels **(C, D)** different dogs were examined.

We used flow cytometry to examine the expression patterns of CD16A and CD64 by dog peripheral blood leukocytes. The general leukocyte populations of polymorphonuclear cells (PMNs), monocytes, and lymphocytes were identified based on their characteristic forward and side light-scattering and by their expression of well characterized markers, including CD4 (neutrophils and lymphocyte), CD14 (monocytes), and CD5 (T cells) (**Figure 2A**). The anti-canine CD16A clone 4A5 stained a subset of monocytes and lymphocytes, and for some dogs it marginally stained PMNs and/or a small subset of cells in this population (**Figure 2A**). This staining pattern was consistent for all the anti-canine CD16 mAbs generated, which had distinct complementarity-determining region nucleotide sequences from 4A5 (data not shown). In contrast to the expression pattern of CD16A, the anti-canine CD64 clone 10 stained essentially all PMNs and monocytes (**Figure 2A**). With some dogs, we noted a small subset of unstained cells in the PMN population (**Figure 2A**). Taken together, the expression patterns of CD16A and CD64 on dog peripheral blood leukocytes were very similar to that of their human orthologues on peripheral blood leukocytes (47).

We next examined dog peripheral blood lymphocytes to determine which subsets expressed CD16A based on available phenotypic markers. To assess its expression on T cells, we used mAbs to canine α/β TCR, γ/δ TCR, CD8, CD4, and CD3. We found that canine CD16A was expressed on T cells, and that CD3^+^ CD16A^+^ T cells (**Figure 2B**, panel 1) represented 3.08% (± 1.94% SD) of the peripheral blood lymphocytes in the group of dogs that were examined (n = 12). Moreover, CD16A^+^ lymphocytes primarily expressed an α/β TCR versus γ/δ TCR (**Figure 2B**, panels 2 and 3) and CD8 versus CD4 (**Figure 2B**, panels 4 and 5). CD16A expression was also observed on CD3^−^ lymphocytes (**Figure 2B**, panel 1) as well as α/β TCR^−^ lymphocytes (**Figure 2B**, panel 2), which usually was a smaller subset than CD3^+^ CD16A^+^ T cells and consisted of 1.23% ± 0.97% SD, n = 12) of the peripheral blood lymphocytes. B cells were identified by their expression of CD20 and CD22 (data not shown), and essentially none of these cells expressed CD16A (**Figure 2B**, panel 6).

In dogs, CD5 is typically classified as a canine T cell marker and is expressed at varying densities, referred to as CD5^dim^ and CD5^bright^ (24). We detected CD16A expression on CD5^−^ and CD5^dim^ lymphocytes, but not on CD5^bright^ lymphocytes (**Figure 2B**, panel 7). In most dogs we observed that CD5^dim^ CD16A^+^ lymphocytes were the predominant population, but in some dogs CD5^−^ CD16A^+^ lymphocytes were an equivalent or the predominant population (**Figure 2C**). Within the CD16A^+^ lymphocyte population, CD5 expression corresponded with CD3 expression. That is, CD16A^+^ lymphocytes were either CD3^+^ CD5^+^ or CD3^−^ CD5^−^ (**Figure 2C**). In humans, the non-B cell, CD3^−^ CD5^−^ CD16A^+^ lymphocyte population represents NK cells (48). CD56 and CD94 are broad markers of human NK cells (49, 50). CD56 is not an NK cell marker in dogs (51, 52), whereas CD94 has been reported to be expressed by dog NK and NK T cells (53). Using a commercially available anti-human CD94 mAb that cross-reacts with canine CD94 (53), we found it stained a portion of CD3^−^ CD16A^+^ and CD3^+^ CD16A^+^ lymphocytes, which varied between dogs (**Figure 2D**). The above findings thus indicate that CD16^+^ lymphocytes in the dog consist of NK cells and T cells (e.g., NK T cells), and that the latter is the predominant population, which contrasts with humans (48, 54, 55).

### Ectodomain Shedding of Canine CD16A

**Figure 3:**
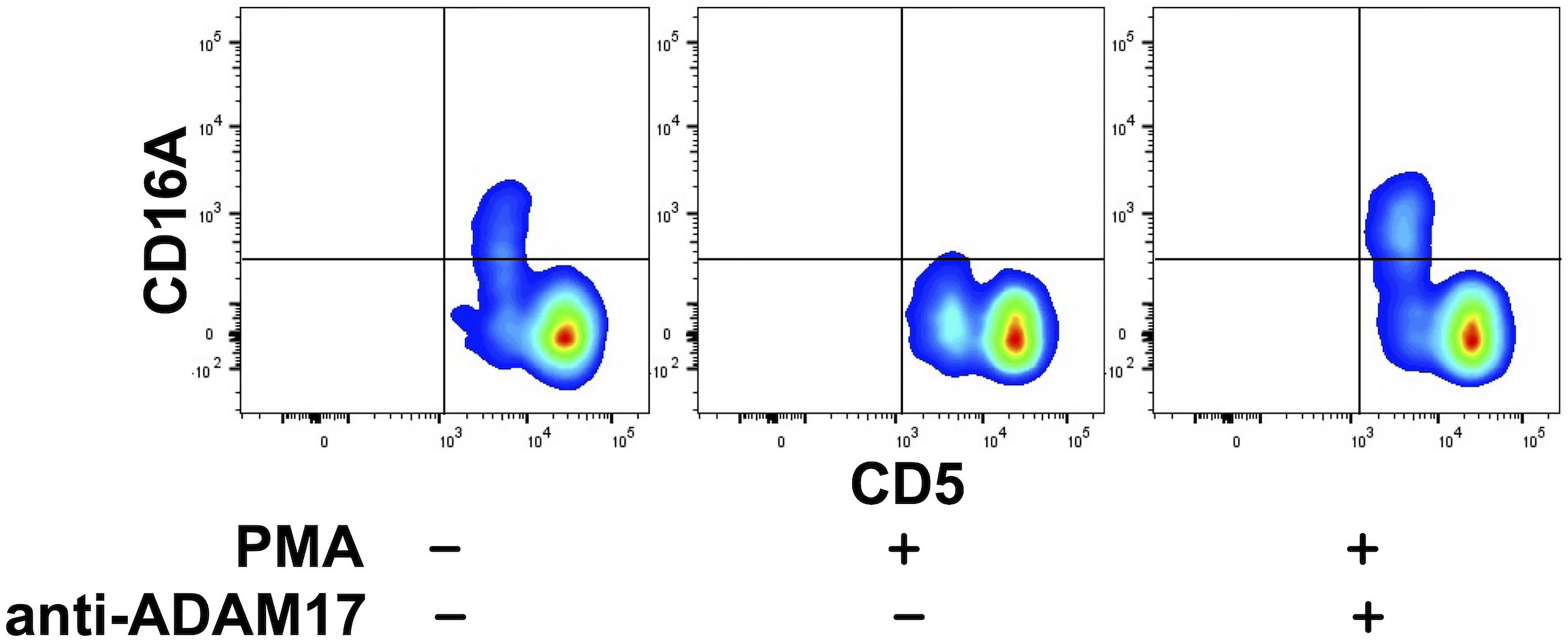
Canine CD16A is downregulated by ADAM17 upon lymphocyte activation. Canine peripheral blood mononuclear cells were treated with or without PMA in the presence or absence of an ADAM17 function blocking mAb. Relative cell-staining levels of CD16A on CD5^dim^ or CD5^bright^ cells were determined by flow cytometry. All density plots show representative data of three independent experiments using leukocytes from separate canine donors.

Human CD16A undergoes a rapid downregulation in expression by a proteolytic process mediated by a disintegrin and metalloproteinase-17 (ADAM17) upon cell activation with various stimuli (23, 40, 56, 57). For instance, the treatment of human leukocytes with the phorbol ester PMA induces efficient CD16A downregulation by ADAM17 (40). We found that this also occurred upon the activation of canine leukocytes with PMA. For instance, in **Figure 3**, CD16A levels on CD5^dim^ lymphocytes are shown before and after PMA activation. We have previously reported on ADAM17 activity in dog neutrophils and that it can be blocked by an ADAM17 mAb (33). This mAb also blocked the downregulation of CD16A on activated canine lymphocytes (**Figure 3**). Taken together, our findings reveal a similar regulation of human and canine CD16A by ADAM17, a process that does not occur for mouse CD16 (23).

### Induction of ADCC by Canine CD16A and CD64

**Figure 4:**
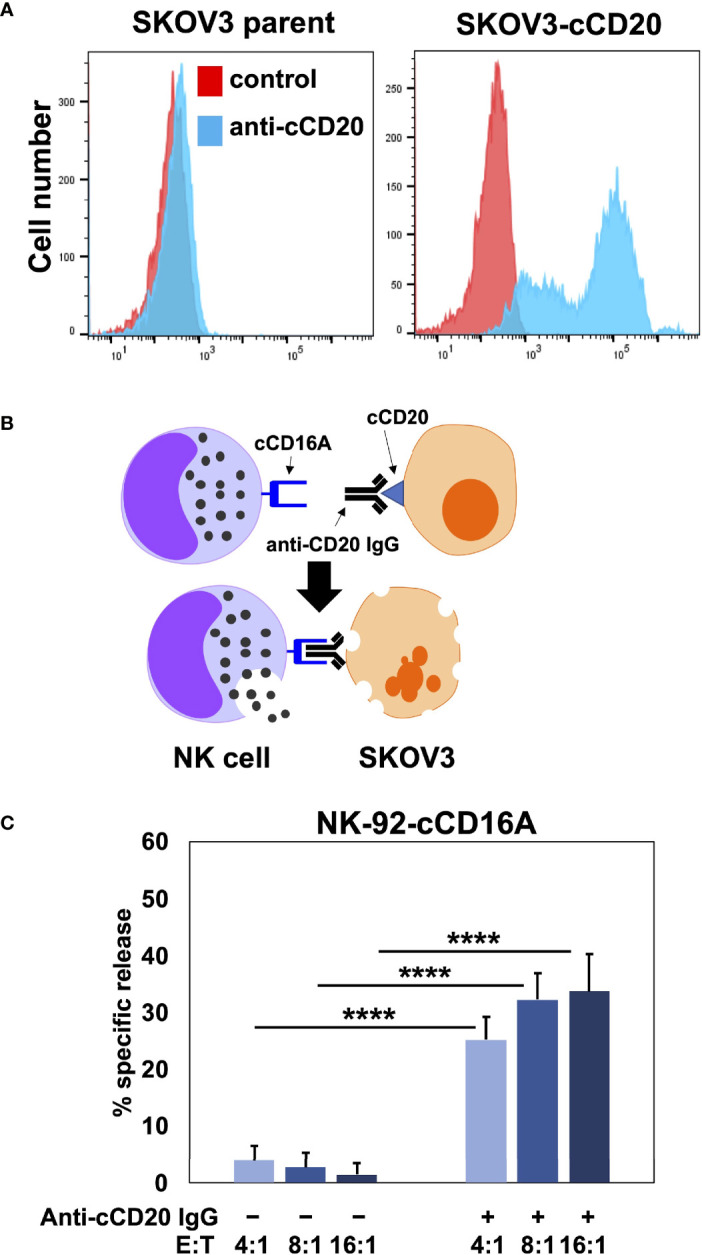
NK-92 cells expressing canine CD16A mediate ADCC. **(A)** SKOV-3 parental cells and SKOV-3-canine CD20 (cCD20) cells were stained with an anti-canine CD20 mAb or an isotype-matched negative control mAb (control) and examined by flow cytometry. **(B)** Schematic representation of ADCC. SKOV-3-canine CD20 cells treated with a caninized anti-canine CD20 mAb in the presence of NK-92 canine CD16A (cCD16A) cells. **(C)** NK-92 canine CD16A cells were incubated with SKOV-3 canine CD20 cells at the indicated E:T ratios in the presence or absence of a caninized anti-canine CD20 mAb for 2 h at 37 °C. Data are represented as % specific release and the mean ± SD of 3 independent experiments is shown. Statistical significance is indicated as ****p < 0.0001.

Human CD16A is a potent activating receptor that induces ADCC upon engaging antibodies attached to target cells (1). We directly tested whether canine CD16A could induce ADCC. For this experiment, NK-92 cells were transduced with intact canine CD16A that lacked an HA-tag due to the potential it might interfere with IgG binding. Canine CD16A expression was verified using the anti-canine CD16A mAb 4A5 (data not shown). To avoid a xenogeneic response between NK-92 cells and canine target cells, as has been reported by others (58), we used the human ovarian cancer cell line SKOV-3 as target cells that we transduced to express canine CD20 (**Figure 4A**). Like human IgGs, canine IgGs consist of four subclasses (IgG 1, 2, 3, and 4 also referred to as A, B, C, and D) (16, 59). Canine IgG2 (IgGB) is the functional analog of human IgG1 and it binds to canine CD16A and canine CD64 (16). To target canine CD20, we used a commercially available “caninized” mAb containing a canine IgG2 Fc region (**Figure 4B**). We observed that NK-92 cells expressing canine CD16A mediated significantly higher levels of cytolysis in the presence of the anti-CD20 mAb when compared to cells in the absence of the mAb at various effector:target (E:T) ratios (**Figure 4C**).

**Figure 5:**
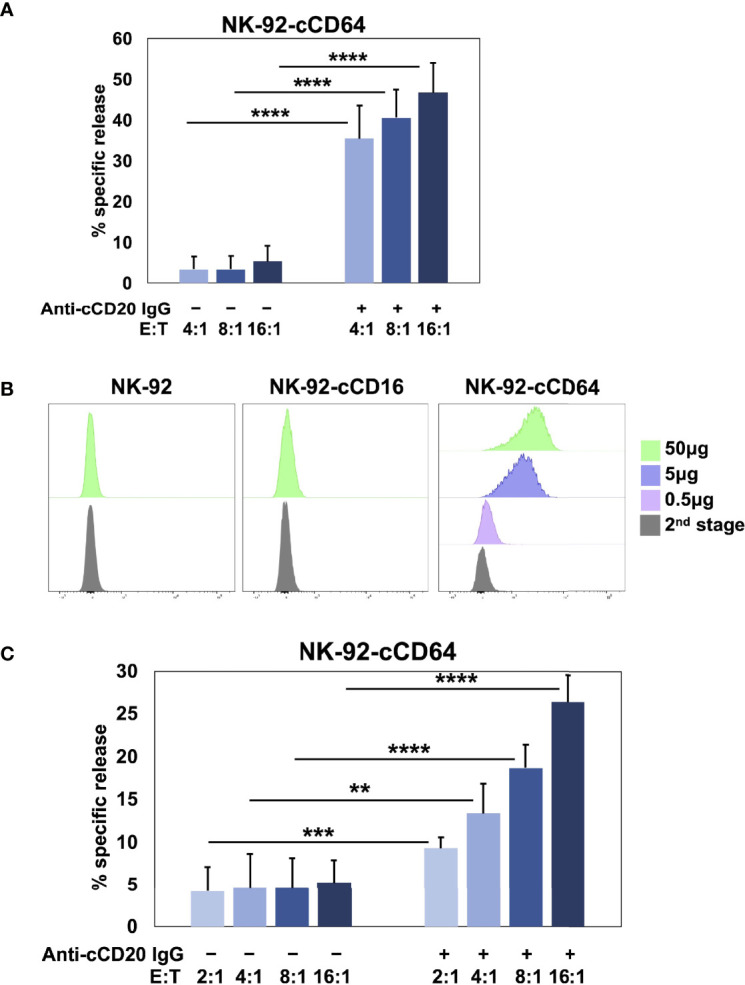
NK-92 cells expressing canine CD64 mediate ADCC when armed with a tumor-targeting mAb. **(A)** NK-92 canine CD64 (cCD64) cells were incubated with SKOV-3 canine CD20 cells at the indicated E:T ratios in the presence or absence of a caninized anti-canine CD20 mAb for 2 h at 37°C. Data are represented as % specific release and the mean ± SD of 3 independent experiments is shown. Statistical significance is indicated as ****p < 0.0001. **(B)** NK-92-cCD16A and NK-92-cCD64 cells were incubated with or without biotinylated canine IgG at various concentrations for 1 h at 37°C, washed, stained with fluorophore-conjugated streptavidin, and analyzed by flow cytometry. Data are representative of at least 3 independent experiments. **(C)** NK-92 canine CD64 cells were incubated in the presence or absence of caninized anti-canine CD20 mAb (5μg/ml), washed, and exposed to SKOV-3 canine CD20 cells at the indicated E:T ratios for 2 h at 37°C. Data are represented as % specific release and the mean ± SD of 3 independent experiments is shown. Statistical significance is indicated as **p < 0.01, ***p < 0.001, ****p < 0.0001.

Human and canine CD16A binds to IgG with low affinity as well as reduced avidity when downregulated in expression by ADAM17 upon NK cell activation. These may serve as checkpoint processes for maintaining immune homeostasis, but they also reduce the efficacy of anti-tumor therapeutic mAbs (3). To address this, we have engineered human NK cells with recombinant versions of CD64 (14, 17, 18), the only high affinity FcγR (10, 60). Human CD64 binds to the same IgG isotypes as CD16A (10), as is also the case for canine CD64 (16), and it signals the same as CD16A by noncovalent association with FcRγ and/or CD3ζ (18). Moreover, CD64 is not cleaved by ADAM17 (17). **Figure 5A** shows that NK-92 cells expressing canine CD64 also effectively mediated ADCC of SKOV-3 canine CD20 cells in the presence of a caninized anti-CD20 mAb. Due to the high affinity state of CD64, we found that engineered NK-92 cells expressing human CD64 could be armed with tumor-targeting mAbs and mediate ADCC (17, 18). NK-92 cells expressing canine CD64 also bound soluble monomeric canine IgG and at higher levels than NK-92 cells expressing canine CD16A (**Figure 5B**). Additionally, NK-92 cells expressing canine CD64 could be armed with a caninized anti-CD20 mAb and mediate ADCC of SKOV-3-cCD20 cells (**Figure 5C**). Collectively, our data demonstrate that canine CD16 and CD64 can induce ADCC by NK-92 cells, similar to their human orthologues.

## Discussion

Human CD64 and CD16A play a critical role in the effector activities of anti-tumor therapeutic mAbs (15). Both FcγRs have been cloned from dog leukocytes and their IgG binding characteristics examined (16). However, their cell surface expression patterns on dog leukocytes have not been previously determined. We generated mAbs to canine CD64 and CD16A and show their expression on myeloid and lymphoid leukocyte subsets in the dog, which in general was similar to humans (47). A closer look at canine lymphocytes revealed CD16A expression on CD3^+^ T cells and CD3^−^ lymphocytes, but not on B cells. Interesting is that canine CD16A^+^ lymphocytes were predominantly in the CD3^+^ fraction, and expressed an α/β TCR and CD8, whereas in humans, CD16A^+^ lymphocytes primarily occur in the CD3^−^ fraction (48). Though, it has been reported that the proportion of α/β TCR^+^ CD8^+^ CD16A^+^ T cells in humans can increase during certain viral infections, and that these cells demonstrated NK cell properties (55). Huang et al., reported that the CD5^dim^ canine lymphocyte population was primarily α/β TCR^+^ and CD8^+^ with NK cell-like effector functions (38). We speculate that canine NK T cells include CD3^+^ CD5^dim^ α/β TCR^+^ CD8^+^ CD94^+^ CD16A^+^ cells, and it will be interesting to examine their effector functions in future studies.

Canine NK cells are phenotypically distinguished based on their lack of B cell markers, CD3, CD4, and TCRs (24, 25). CD5^bright^ lymphocytes in the dog have been classified as T cell (24); however, there are conflicting views on CD5 expression by canine NK cells, which have been reported as CD5^+/−^ and CD5^dim^ (27, 30, 38). Both populations have been shown to mediate natural cytotoxicity and ADCC (27, 28, 30, 31, 38). We observed that CD5 expression levels on CD16A^+^ lymphocytes ranged from negative to dim, and this directly corresponded with their CD3 expression. Thus, canine CD16A^+^ lymphocytes appear to consist of CD3^+^ CD5^dim^ T cells, as discussed above, and CD3^−^ CD5^−^ CD20^−^ (or CD22^−^) lymphocytes. The phenotypic characterization of canine NK cells is at this point is a work in progress. NK cell markers detected by antibodies consist of CD94 and NKp46 (24). We show that CD94 expression varied on CD3^−^ CD16A^+^ lymphocytes, as well CD3^+^ CD16A^+^ lymphocytes. NKp46 expression has been reported to also vary on dog peripheral blood NK cells, and its expression appears to indicate recent activation and expansion (27, 29, 31, 61, 62). Using the only commercially available anti-canine NKp46 mAb, we observed inconsistent staining of dog peripheral blood lymphocytes, which at times did not stain above an isotype-matched negative control mAb (data not shown). As is the case in humans (1), CD16A may represent a pan-marker of mature NK cells in the peripheral blood of dogs.

Our study shows that the clone 4A5 mAb recognizes canine CD16A and not CD64. CD32B is the only other expressed canine FcγR (16). We did not directly demonstrate that 4A5 does not recognize canine CD32B; however, we feel this is unlikely considering that full-length canine CD16A and CD32B have only ~30% amino acid identity (data not shown). In addition, CD32B is expressed by human and mouse B cells and functions as an inhibitory receptor that blocks antibody production (10). We did not observe 4A5 staining of canine B cells.

Mouse leukocytes express in addition to classic CD16, CD16-2 (FcγRIV), an orthologue of human CD16A (10, 60). A review article by Nimmerjahn et al. in 2006 reported on the presence of an *FCGR4* gene on chromosome 38 in the dog (19). This was based on an early rough draft of the dog genome sequence from 16 years ago. According to the most updated *Canis lupus familiaris* assembly ROS_Cfam_1.0 (RefSeq: GCF_014441545.1), chromosome 38 of C. lupus (updated on December 23, 2021, Chr 38_21,137,358.21,146,800, Length: 9,443 nucleotides) contains the *FCGR3A* gene (also known as LOC478984), which encodes for the low affinity IgG Fc receptor CD16A, and lacks the *FCGR4* gene. Humans also lack this gene (10, 60).

We directly show that canine CD16A when expressed in the human NK cell line NK-92 can induce ADCC. Others have reported that NK-92 cells expressing a canine CD16A fusion protein consisting of the extracellular region of canine CD16A and the transmembrane and intracellular regions of canine FcRγ also mediated ADCC (58). Human CD16A signaling is normally mediated by a non-covalent association with the signaling adapters FcRγ and/or CD3ζ (1, 63, 64). NK-92 cells express FcRγ and CD3ζ (65), and therefore canine CD16A likely associated with these signaling adaptors to induce ADCC. Indeed, seven amino acids in the transmembrane region of human CD16A have been shown to be critical for FcRγ and CD3ζ association (65), and all seven amino acids are conserved in the transmembrane region of canine CD16A (**Supplementary Figure 1A**).

A critical effector function of several clinically successful mAbs targeting tumors is ADCC (66); however, CD16A undergoes rapid shedding from the cell surface of NK cells by ADAM17 following its signaling or by various other stimuli (23, 40, 56, 57). This appears to reduce the efficacy of tumor mAb therapies, and perhaps especially so in the tumor microenvironment (3). Indeed, CD16A downregulation has been reported to occur by NK cells in the tumor microenvironment of ovarian cancer (67). ADAM17 activity has been demonstrated in canine leukocytes (33), and we show in this study that blocking the metalloprotease prevented CD16A downregulation upon canine lymphocyte activation. Further studies will be required to determine if canine CD16A is cleaved at the same location as human CD16A and the functional effects of blocking this process. Human NK cells expressing a non-cleavable version of CD16A or upon blocking ADAM17 activity demonstrated increased killing of tumor cells by ADCC and production of IFNγ (5–7), and thus this may be an approach to enhance ADCC by CD16A^+^ canine lymphocytes.

Another property of CD16A that appears to decrease the efficacy of mAb therapies is that it is a low affinity member of the FcγR family. Indeed, clinical studies in humans have shown that increasing the affinity by which NK cells engage anti-tumor mAbs significantly improved patient outcome (11, 12, 68, 69). This is an active area of research that includes approaches such as modifying the Fc region of anti-tumor antibodies or the FcγR on NK cells (14, 15, 66). For the latter, we have engineered human NK cells to express recombinant versions of human CD64 (17, 18), the only high affinity IgG Fc receptor in humans and dogs (10, 16). Human CD64 is normally expressed by myeloid cells and not NK cells (10). We observed the same CD64 expression pattern in dogs. Similar to canine CD16A, canine CD64 expression in NK-92 cells induced ADCC, and due to its high affinity state, monomeric canine IgG could be adsorbed to these cells. Additionally, NK-92 canine CD64 cells armed with a caninized anti-canine CD20 mAb effectively killed target cells expressing canine CD20. NK-92 canine CD64 cells appeared to have higher ADCC activity when anti-canine CD20 mAb was added to the assay compared to NK-92 canine CD64 cells armed with anti-canine CD20 mAb; however, we did not perform a direct comparison. Nevertheless, it is possible that some of the adsorbed mAb internalized (especially if antibody aggregates were present) or dissociated from CD64, leading to lower ADCC activity by the armed NK-92 canine CD64 cells. It will be interesting to determine in future studies if primary dog NK cells or T cells can be engineered to express canine CD64 and if this enhances their ADCC potency. NK cells, due to their lack of graft-vs-host response, expressing canine CD64 could provide an off-the-shelf adoptive cell therapy option used in combination with assorted anti-tumor targeting mAbs. This would provide broad tumor antigen targeting to reduce tumor antigen escape and for treating various malignancies. The development of mAbs that target tumor antigens in dogs and induce ADCC has been minimal at this time (70). However, as tools and our understanding about canine immune cells rapidly increase, it is likely a surge in immunotherapies for dogs will follow.

Dogs also offer a key animal model for the translation of immunotherapies. The study of ADCC in rodent syngeneic tumor models is limited by species differences in immune cell effector functions, including significant differences in human and mouse CD16 expression and function (see Introduction). The dog spontaneous cancer model incorporates heterogeneous disease and an intact immune system with many similarities to humans to help determine the mechanisms that underlie success and failure of immunotherapies (24–26). We have extended the current understanding of the dog immune system by demonstrating phenotypic and functional characteristics of canine CD16A and CD64 to advance utilizing these FcγRs for therapeutic approaches.

## Data Availability Statement

The raw data supporting the conclusions of this article will be made available by the authors, without undue reservation.

## Ethics Statement

The animal study was reviewed and approved by Institutional Animal Care and Use Committee of the University of Minnesota. Written informed consent was obtained from the owners for the participation of their animals in this study.

## Author Contributions

RH, YL, KS, SW, CL, CZ, and CF designed the experiments, collected, assembled, analyzed, and interpreted the data. JW and BW designed the experiments, interpreted the data, and wrote the manuscript. PM contributed vital reagents. JM and AB interpreted the data. All authors contributed to the manuscript preparation as well as read and approved the submitted version.

## Funding

This work was supported by University of Minnesota College of Veterinary Medicine Comparative Medicine Signature Program Grant (to BW, JW, AB); University of Minnesota Animal Cancer Care and Research Program Canine Cancer Charitable Grant and GreyLong Gift (to BW); Howard Hughes Medical Institute and Burroughs Wellcome Fund Medical Research Fellowship (to KS); NIH UL1TR002494 supporting the University of Minnesota Clinical and Translational Science Institute K-R01 Scholar Award (to AB), and NIH P30 CA77598 supporting the University of Minnesota, Masonic Cancer Center, Flow Cytometry resource.

## Conflict of Interest

The authors declare that the research was conducted in the absence of any commercial or financial relationships that could be construed as a potential conflict of interest.

## Publisher’s Note

All claims expressed in this article are solely those of the authors and do not necessarily represent those of their affiliated organizations, or those of the publisher, the editors and the reviewers. Any product that may be evaluated in this article, or claim that may be made by its manufacturer, is not guaranteed or endorsed by the publisher.

## Supplementary Material

Supplementary Figure 1 Amino acid alignment of human and canine CD16A or canine CD64. The amino acid sequences of human and canine CD16A and CD64 are from the NCBI reference sequences (human CD16: X52645.1; canine CD16A: XM_022415348; human CD64: X14356.1; canine CD64: NM_001002976). The amino acid sequence of the human CD16A and CD64 transmembrane regions (underlined) is based on a previous study (71). **(A)** Alignment of human and canine CD16A. The red boxed letters represent amino acids critical for association with the signaling adaptors FcRγ and CD3ζ (65). **(B)** Alignment of human and canine CD64.
