# Supplementary figures and images for: Examination of IgG Fc Receptor CD16A and CD64 Expression by Canine Leukocytes and Their ADCC Activity in Engineered NK Cells
Source: Front Immunol. 2022 Feb 24;13:841859. doi: 10.3389/fimmu.2022.841859 (PMC8907477; doi:10.3389/fimmu.2022.841859)

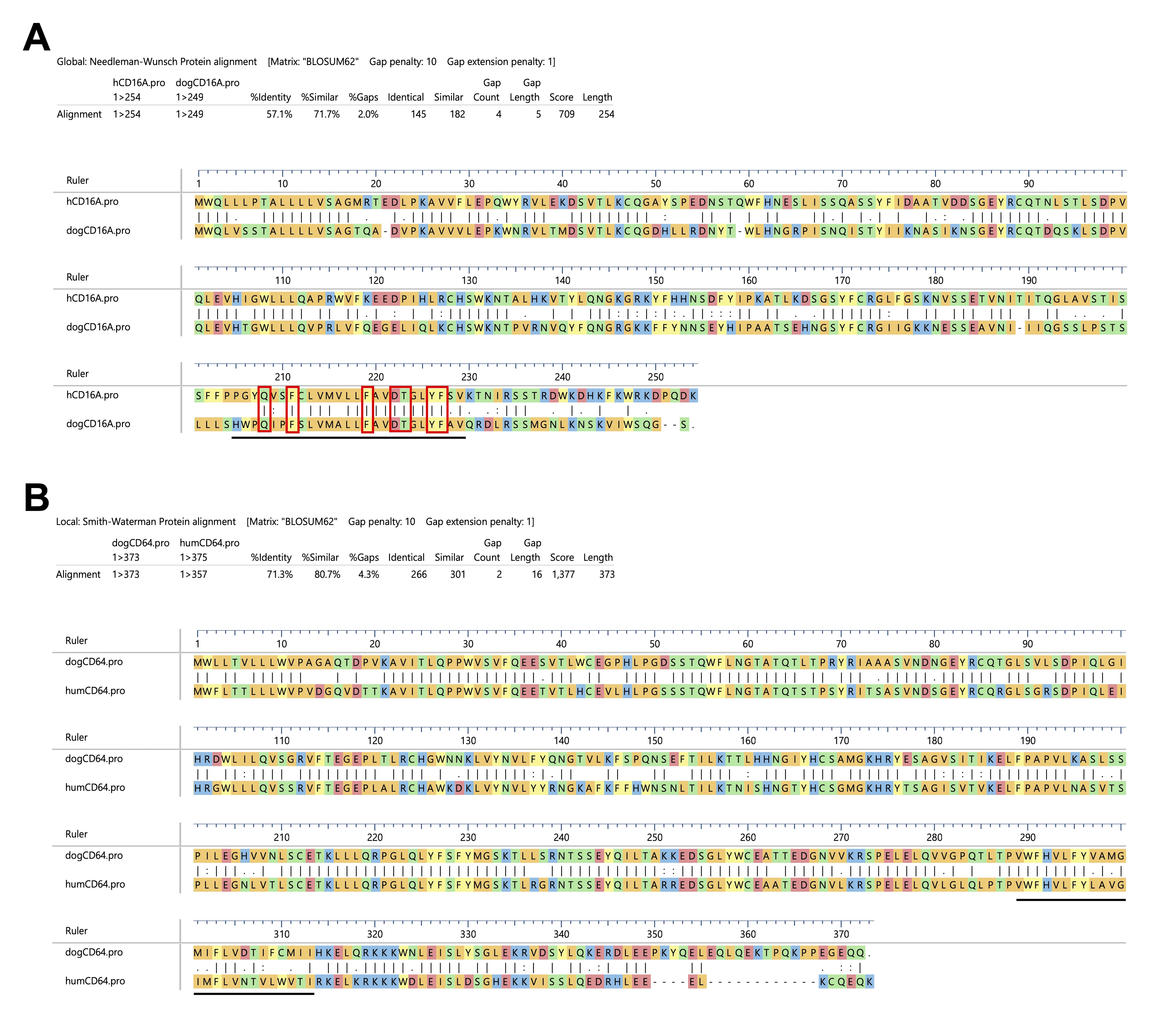

Supplement: Supplementary Figure 1 — Amino acid alignment of human and canine CD16A or canine CD64. The amino acid sequences of human and canine CD16A and CD64 are from the NCBI reference sequences (human CD16: X52645.1; canine CD16A: XM_022415348; human CD64: X14356.1; canine CD64: NM_001002976). The amino acid sequence of the human CD16A and CD64 transmembrane regions (underlined) is based on a previous study (71). (A) Alignment of human and canine CD16A. The red boxed letters represent amino acids critical for association with the signaling adaptors FcRγ and CD3ζ (65). (B) Alignment of human and canine CD64. [file Image_1.jpeg]
